# Conservation of the Exon-Intron Structure of Long Intergenic Non-Coding RNA Genes in Eutherian Mammals

**DOI:** 10.3390/life6030027

**Published:** 2016-07-15

**Authors:** Diana Chernikova, David Managadze, Galina V. Glazko, Wojciech Makalowski, Igor B. Rogozin

**Affiliations:** 1 Department of Genetics, Institute for Quantitative Biomedical Sciences, Geisel School of Medicine, Dartmouth College, Hanover, NH 03755, USA; dchernikova@gmail.com; 2 Information Engineering Branch, National Center for Biotechnology Information, National Library of Medicine, National Institutes of Health, Bethesda, MD 20894, USA; dmanagadze@gmail.com; 3 Department of Biomedical Informatics, University of Arkansas for Medical Sciences, Little Rock, AR 72205, USA; GVGlazko@uams.edu; 4 Institute of Bioinformatics, University of Muenster, Muenster 48149, Germany; wojmak@uni-muenster.de; 5 Computational Biology Branch, National Center for Biotechnology Information, National Library of Medicine, National Institutes of Health, Bethesda, MD 20894, USA; 6 Novosibirsk State University, Novosibirsk 630090, Russia

**Keywords:** lincRNA, exon, intron, non-coding RNA, genomic alignments, intron gain, intron loss

## Abstract

The abundance of mammalian long intergenic non-coding RNA (lincRNA) genes is high, yet their functions remain largely unknown. One possible way to study this important question is to use large-scale comparisons of various characteristics of lincRNA with those of protein-coding genes for which a large body of functional information is available. A prominent feature of mammalian protein-coding genes is the high evolutionary conservation of the exon-intron structure. Comparative analysis of putative intron positions in lincRNA genes from various mammalian genomes suggests that some lincRNA introns have been conserved for over 100 million years, thus the primary and/or secondary structure of these molecules is likely to be functionally important.

## 1. Introduction

Recent years witnessed rapidly-growing interest in long non-coding RNAs (lncRNAs), a relatively new performer on the genomic stage. However, despite many efforts, lncRNAs still hold a status of the genomic ‘dark matter’ [1,2]. Indeed, while other non-coding RNA molecules (ribosomal, transfer, small nuclear, antisense, small nucleolar, micro, and Piwi-interacting RNAs) have already been assigned well-defined functional roles, the functions of lncRNAs remain largely unknown [2,3,4,5]. Even their definition is somewhat vague: lncRNAs are defined as non-coding transcripts longer than ~200 nucleotides [1]. A popular view that the vast majority of long intergenic non-coding RNAs (lincRNAs) are byproducts of background transcription, “simply the noise emitted by a busy machine” [6,7], is rooted in their typically low abundance and poor evolutionary conservation compared to protein-coding sequences and small RNAs such as miRNAs and snoRNAs [8]. However, some of the lncRNAs contain evolutionary conserved regions [9], and most lncRNAs show reduced substitution and insertion/deletion rates suggestive of purifying selection [10,11,12,13,14]. While the number of lncRNAs may be large [15,16], the combination of various experimental approaches applied to transcriptomes of several species has resulted in continuous discovery of new transcripts, with the FANTOM project alone cataloguing more than 30,000 putative long non-coding transcripts in mouse tissues by full-length cDNA cloning [17]. Although the sequences of most lncRNAs are much less conserved than protein sequences, the extent of orthology between the lncRNomes is unexpectedly high, with 60% to 70% of the lncRNA genes shared between human and mouse [18].

Most lncRNAs display specific subcellular localization and are processed (polyadenylated and spliced); this observation argues that they most likely function in their mature form [2,19]. Another indication that lncRNA products may be functional is that much of the evolutionary constraint on lncRNA sequence is likely to be localized at splicing regulatory elements [20,21], suggesting that correct splicing of introns is important for function. Indeed, the vast majority of lncRNAs with demonstrated cellular function (functional lncRNAs) appear to act as processed RNAs [2,19]. Comparative analysis of more than 3,000 mouse lncRNA genes suggested that conservation of the exon-intron structure might be a general lncRNA property [10]. It was found that 65% and 40% of mouse lncRNA |GT-AG| splice sites are conserved in rat and human, respectively. These numbers are significantly greater than the number of conserved intronic GT and AG dinucleotides that are not involved in splicing, indicating evolutionary conservation of splice signals in lncRNAs [10].

Among the transcripts are numerous long intergenic non-coding RNAs (lincRNAs), i.e., RNA molecules greater than 200 nucleotides in length that are encoded outside other identified genes. One of the best studied lincRNAs is *Xist*, which is involved in X-chromosome inactivation in females of eutherian mammals [22,23]. The *Xist* RNA appears to have evolved as a result of the *Lnx3* protein-coding gene losing its protein coding ability and becoming a pseudogene in early eutherians, followed by integration of mobile elements [24,25]. Four of the ten *Xist* exons found in eutherians show significant sequence similarity to exons of the *Lnx3* gene, whereas the remaining six *Xist* exons are similar to different transposable elements. Thus, some *Xist* introns were inherited from the *Lnx3* gene, but some appear to have been gained in the course of evolution of the *Xist* gene [25]. Analysis of *Xist* in several mammalian species revealed an overall conservation of the *Xist* exon-intron structure [25].

Here we attempted large-scale reconstructions of the evolution of introns in lincRNA genes using multiple genomic alignments. Comparative analysis of putative intron positions in lincRNA genes from various mammalian genomes suggests that some lincRNA introns have been conserved for over a hundred million years, and thus the primary/secondary structure of these molecules is likely to be functionally important.

## 2. Materials and Methods

Human and mouse lincRNA genes, the corresponding genomic alignments, and expression data were taken from our previous work [13] where the procedures of data processing are described in full detail. Briefly, the dataset of 5444 “Noncoding Only” mouse probe sets were downloaded from the NRED database [26]. After discarding the probe sets that did not map to intergenic regions and establishing one-to-one relationship between RNA IDs and their corresponding probe set IDs, we obtained the final set of 2390 mouse lincRNAs (NCBI GenBank Accession IDs of RNAs) of which 977 contained introns. After discarding the probe sets with very low median expression levels, as well as those with equivocal genome mapping, the final set of 2013 mouse lincRNAs, including 918 intron-containing ones, was obtained. For humans, the data for 917 probe sets were downloaded, and the same procedure of removing low-expressed or equivocally mapped lincRNAs yielded the final set of 519 lincRNAs including 211 intron-containing genes. The genomic coordinates and sequences of exons and introns of human and mouse lincRNA genes were downloaded from the UCSC Table Browser [27], from “all_mrna” tables of mouse mm8 and human hg18 assemblies. Multiple alignments of these regions were fetched from the Galaxy system [28]. Two different 17-way multiZ alignments were employed (with human (hg18) and mouse (mm8) reference genomes). The following species were used for our analysis: human (hg18), chimp (panTro1), cow (bosTau2), macaque (rheMac2), mouse (mm8), rat (rn4), dog (canFam2), tenrec (echTel1), elephant (loxAfr1), rabbit (orCun1), zebrafish (danRer3), opossum (monDom4), armadillo (dasNov1), chicken (galGal2), fugu (fr1), tetraodon (tetNig1), and frog (xenTro1) [13]. Parsimony analysis was performed using the DNAPARS program from the PHYLIP package. In order to test significance of evolutionary conservation of splicing signals (GT or GC (introns start) and AG (intron end) dinucleotides) and intron positions, we estimated the fraction of conserved splicing signals (F_real_). After this we randomly sampled GT/GC (or AG) dinucleotides from alignments of intronic sequences and evaluated the fraction of conserved GT/GC (or AG) dinucleotides (F_sampled_). We repeated the sampling procedure 10,000 times, the distribution of F_sampled_ was used to calculate probability *P* (F_real_ ≤ F_sampled_). This probability is equal to the fraction of sampled splicing signals (GT/GC or AG) in which F_sampled_ is the same or higher than F_real_. Small probability values *P* (F_real_ ≤ F_sampled_) ≤ 0.05 indicate a significant conservation of splicing signals. The same procedure was repeated for introns, and in this case the conservation of GT/GC and AG dinucleotides was studied simultaneously. The distance between GT/GC and AG was required to be greater than 39 nucleotides, as suggested by Deutsch and Long [29]. Observed distributions of human and mouse intron lengths (Table 1) and frequencies of GT/GC dinucleotides were simulated during the sampling procedure. The fraction of conserved donor and acceptor splicing signals GT-AG and GC-AG was used to calculate the probability *P* (F_real_ ≤ F_sampled_).

## 3. Results

### 3.1. Datasets

We sought to analyze the evolution of intron-exon structure of mammalian lincRNA genes on the scale of complete genomes. Such an analysis requires careful identification of orthologous gene sets (sets of genes derived from a single ancestral gene in the last common ancestor of the compared species) as well as identification of orthologous (“the same”) introns in each of these gene sets. To avoid potential complications caused by coordinated expression of protein-coding genes and lncRNAs, we chose to analyze only the sets of mammalian lincRNAs. We used human and mouse datasets because these curated lincRNA sets have known evolutionary and gene expression properties [13,18]. This dataset is unlikely to contain protein-coding genes [13,18], and this same conclusion was reached for other datasets of lincRNA genes [30,31]. The smaller sample size of human lincRNA genes compared to mouse lincRNA genes (Table 1) did not perceptibly affect the conclusions of several previous studies [13,14,18]. The characteristics of the sets of mouse and human lincRNAs analyzed here are summarized in the Table 1. Approximately 40% human and mouse lincRNAs contain introns (Table 1). There are more than 2 introns per intron-containing lincRNA genes with the average intron length more than 9,000 nucleotides, although median values are much smaller (Table 1). Interestingly, despite of longer exons in mice than humans and similar intron size in both species, the average length of mouse lincRNAs is significantly shorter than the average length of human lincRNAs (the two-tailed *P* value is less than 0.0001 according to the Student t-test). This is apparently due to the higher number of introns present in human lincRNAs than in mouse lincRNAs (3.86 compared to 2.52 on average) (Table 1). This result may reflect differences in lincRNA sampling procedures although biological trends should not be excluded.

### 3.2. Evolutionary Conservation of Splicing Signals

We analyzed the evolutionary conservation of GT/GC (introns start) and AG (intron end) using pairwise comparison between mouse/human lincRNA genes and 15 other species (Table 2). In accord with the previous study [10], we found a significant conservation of splicing signals (Table 2). Pairwise comparisons with mouse splicing signals suggested that the fraction of conserved GT/GC and AG dinucleotides in rat is 73% and 68%, respectively. The fraction of conserved GT/GC and AG was around 50%–60% for most comparisons. These numbers are significantly greater than the number of conserved intronic GT/GC and AG dinucleotides that are not involved in splicing indicating evolutionary conservation of splice signals in lncRNAs (*P* (F_real_ ≤ F_sampled_) < 0.001, see the Experimental section). This result suggests that mouse lincRNA genes contain evolutionary conserved splicing signals. However the fraction of conserved GT/GC and AG dinucleotides is much larger (around 70%–80%) for comparisons between human lincRNA introns and orthologous positions in other species (Table 2), the conservation level is highly significant (*P* (F_real_ ≤ F_sampled_) < 0.001).

### 3.3. Evolutionary Conservation of the Exon-Intron Structure

Traditionally, analysis of intron positions in protein-coding genes was based on orthologous intron positions. For a pair of introns to be considered orthologous, they were required to occur in exactly the same position in the aligned sequences of orthologous protein-coding sequences. In this study we used a relaxed definition of orthologous introns based on the whole genome alignments: for a pair of introns to be considered orthologous, one intron needs to be located within known human or mouse lincRNA gene (Table 1) and another one needs to have orthologous GT/GC (introns start) and AG (intron end) dinucleotides in the orthologous positions in at least one sequence from genomic alignments. Thus, we used positions of mouse or human introns as the reference gene structure. This procedure is likely to produce false positives because some dinucleotides may remain conserved but they do not serve as splicing signals. The same problem exists for splicing signals (see above), we used statistical tests to confirm a significant conservation. We applied the same methodology for inferred conserved intron positions in lincRNA genes.

**Figure 1:**
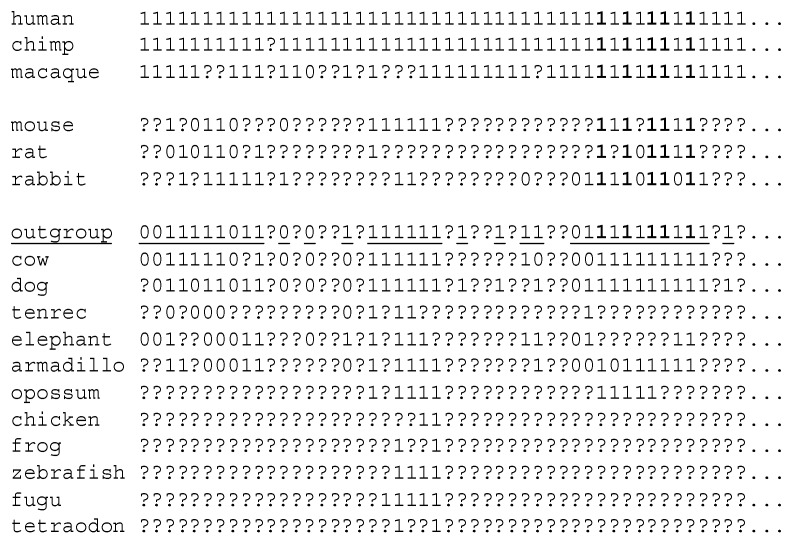
Example of the intron presence/absence matrix. 100% conserved introns are shown in bold. Introns that were used for phylogenetic reconstructions (conserved introns) are underlined in the out-group sequence.

**Figure 2:**
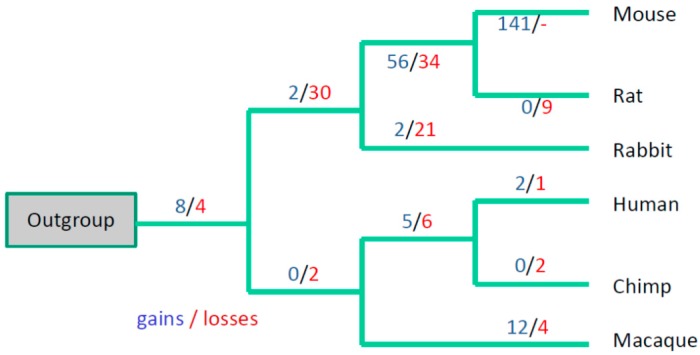
Evolution of exon-intron structure in six Glires and primate species using positions of mouse introns as the reference gene structure. A total of 363 intron positions from mouse lincRNA genes were used for this reconstruction. A total of 124 conserved intron positions (intron positions that were present in the mouse lincRNA genes and in the orthologous position of primates and/or out-group consensus sequence, underlined in the Figure 1) were found. “-” means that loss of introns in mouse cannot be detected because mouse introns were used as a reference in our reconstructions.

**Figure 3:**
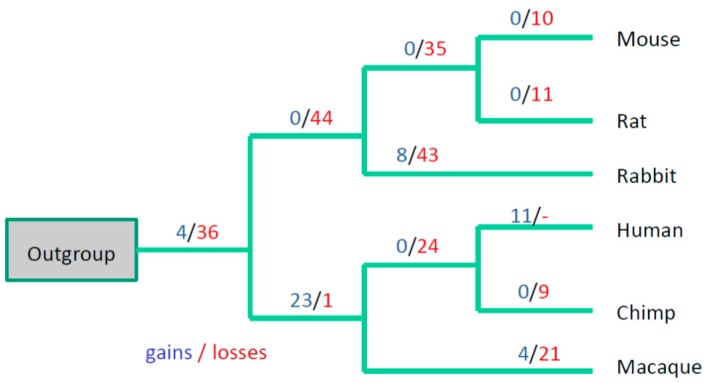
Evolution of exon-intron structure in six primate and Glires species using positions of human introns as the reference gene structure. A total of 656 intron positions from human lincRNA genes were used for this reconstruction. A total of 509 conserved intron positions (intron positions that were present in the mouse lincRNA genes and in the orthologous position of Glires and/or outgroup consensus sequence, underlined in the Figure 1) were found. “-” means that loss of introns in human cannot be detected because human introns were used as a reference in our reconstructions.

To analyze the evolutionary dynamics of introns in greater detail, we turned to phylogenetic analysis. For this purpose, intron positions can be represented as a data matrix of intron absence/presence (encoded as 0/1, missing data is encoded as “?”). An example of such a matrix for intron locations is shown in the Figure 1. We used three primate species and three Glires species as in-groups (that diverged less than 100 million years ago [25]) and the other 11 species as an out-group (Figure 2 and Figure 3). An out-group consensus sequences was reconstructed using the following three rules. (1) If there was at least one “1” in the out-group species, the out-group state was assigned “1” (Figure 1). (2) If there were only “0”s in the out-group species, the out-group state was assigned “0” (Figure 1). (3) If there was no “1” or “0” states in the out-group species, the out-group state was assigned “?” (Figure 1) and this position was removed from further analysis. An example of a fragment of the out-group consensus sequence is shown in the Figure 1. We studied introns that were present in the human lincRNA genes and in at least one of any other species. We used the same filter for mouse lincRNA genes (orthologous introns in mouse lincRNA genes and in at least one of any other species).

The intron absence/presence data were subjected to evolutionary parsimony analysis and, of the existing parsimony approaches, unweighted parsimony seems to be most appropriate in this case because we do not have a model for intron gain/losses in lincRNA genes. We applied the parsimony principle in the following way: given a species tree topology, construct the most parsimonious scenario for intron evolution, i.e., the distribution of intron gain and loss events over the tree branches. The most parsimonious scenario will be the one with the minimal number of gains and losses (Figure 2 and Figure 3).

Analysis of intron positions using the DNAPARS program (see the Experimental section) suggested that many intron positions remained conserved; for example, there are five 100% conserved intron positions in the Figure 1 (we require the 100% conserved intron to be present in all six primate/Glires species and in the out-group consensus sequence). 362 (55%) human intron positions are 100% conserved, the conservation is significant (*P* (F_real_ ≤ F_sampled_) < 0.001). The number of 100% conserved mouse intron positions is less impressive (68 introns, 19%) but still highly significant (*P* (F_real_ ≤ F_sampled_) < 0.001). However, a substantial fraction of mouse and human introns are not conserved (for example, Figure 1). For mouse introns there is a massive intron turnover in the branch leading to mouse-rat clade and in the branch leading to mouse (Figure 2). A similar trend was observed when we used positions of human introns as the reference gene structure (Figure 3), although losses dominated over gains in this scenario.

## 4. Discussion

The apparent paradox of a smaller number of conserved mouse introns compared to human introns (although the mouse lincRNA dataset is much larger, see Table 1) may be a result of a high turnover rate of lincRNA genes/introns in the Glires lineage (Figure 2 and Figure 3). This is consistent with a low conservation of mouse splicing signals compared to human splicing signals (Table 2). These results may reflect the observed fast turnover of rodent lincRNA genes: it was shown that nearly half of lincRNA loci have been gained or lost since the last common ancestor of mouse and rat [32]. It was suggested that such a rapid lincRNA turnover contributes to the evolution of tissue- and lineage-specific gene expression [32]. Frequent losses of introns were observed in several evolutionary conserved lincRNA genes [20], thus the exon-intron structure of lincRNA genes showed considerably greater gain and loss during evolution, whereas comparative analysis of intron positions in protein-coding genes from vertebrates revealed only a few losses but no apparent gain of introns in mammalian genes [33,34]. Larger sets of reliable mammalian lincRNA genes could help to design reliable statistical models of intron gain/loss process and verify any lineage-specific features of this process in various vertebrate lineages.

The substantial turnover of intron positions in mammalian lincRNA genes should not overshadow the observation that many lincRNA introns are remarkably conserved (19%–55%). This observation is consistent with previous studies of the *Xist* gene and several other evolutionary conserved lincRNA genes [20,25]. The present analysis pushes the origin of numerous spliceosomal lincRNA introns back to the radiation of eutherian mammals, approximately 100 million years ago [25]. This result suggests that the primary/secondary structure of these molecules is functionally important and conserved introns can be used as hallmarks of functional lincRNA genes.

It has been suggested that datasets of lincRNA genes do not contain many protein-coding genes [13,18,30,31,35]; however, we cannot exclude the presence of functional short open reading frames [36,37]. One possible indication that lincRNAs do not contain many protein-coding regions is a high fraction of transposable elements observed in lincRNA genes [14]. lincRNAs have twice as many transposable elements as 3’UTRs of protein-coding genes. In fact, the fraction of transposable elements is closer to intronic regions than to any other regions of protein-coding genes [14]. The lower substitution rates of exons compared to introns was observed for human and mouse lincRNA genes [13]. However, purifying selection on the exons in lincRNAs is much weaker than on non-synonymous positions in protein-coding genes [13]. Both the strength and the shape of the distribution of the substitution rates in lincRNA exons more closely resemble synonymous than non-synonymous substitutions in protein-coding genes [13]. This observation is also consistent with the idea that lincRNA are not coding for proteins. However, it cannot be ruled out that the presence of highly conserved introns may be associated with short (and rare) open reading frames. In this case introns can be used as a hallmark of functional short open reading frames. The conclusive answer to this question could be reached by a combination of experimental and computational techniques including ribosome profiling, analysis of codon usage and codon conservation [35]. Alternative splicing is yet another factor that could influence conclusions of this study. In a recent detailed study, over 8,000 human lncRNA genes have been identified, with a mean intron density of ~1.9 per kilobase, and extensive alternative splicing of these non-coding RNAs has been detected, with ~2.3 RNA isoforms per gene [38]. Such alternatively-spliced lincRNAs are likely to increase rates of intron gain/losses.

## 5. Conclusions

We present large-scale reconstructions of the evolution of introns in lincRNAs using multiple genomic alignments of 17 vertebrate species. Comparative analysis of putative intron positions in lincRNA genes from these vertebrate genomes indicates that some lincRNA introns have been conserved for over 100 million years, suggesting that these molecules are likely to be functionally important.

## Figures and Tables

**Table 1:** Statistics of lincRNA datasets.

Features of lincRNA Genes	Mouse	Human
Number of all lincRNAs	2,390	589
Number of intron-containing lincRNAs	979	245
Number of exons	3,439	1,194
Number of introns	2,462	949
Number of exons shorter than 15 nt	41	7
Number of introns per lincRNA	2.52	3.86
Average gene length, nt (standard error)	11,775 (712)	17,192 (1,921)
Median gene length, nt	2,535	2,626
Average exon length, nt (standard error)	524 (21)	409 (48)
Median exon length, nt	464	356
Average intron length, nt (standard error)	9,621 (1,631)	10,562 (4,539)
Median intron length, nt	2,615	2,116

**Table 2:** Conservation of splicing signals (pairwise comparisons between mouse or human and other vertebrates). The number of (putative) orthologs is the number of mouse/human lincRNAs that have an orthologous sequence in other species with the total alignment length ≥ 200 nucleotides. Number of mismatches is the number of dinucleotides different from GT/GC (donor sites) or AG (acceptor sites) in the orthologous positions of alignments.

Species	Common Name (Number of Orthologs)	Splice Site Pairwise Comparison with Mouse or Human as a Reference					
		Donor Splicing Site (GT or GC dinucleotide)			Acceptor Splicing Site (AG dinucleotide)		
		Number of Matches	Number of Mismatches	Percent Matches	Number of Matches	Number of Mismatches	Percent Matches
**Mouse as a reference**							
*Rattus norvegicus*	Rat (2285)	1555	569	73%	1448	669	68%
*Oryctolagus cuniculus*	Rabbit (1522)	518	258	67%	419	306	58%
*Homo sapiens*	Human (2091)	902	619	59%	746	715	51%
*Pan troglodytes*	Chimp (2068)	826	606	58%	703	692	50%
*Macaca mulatta*	Macaque (1971)	807	543	60%	682	647	51%
*Bos taurus*	Cow (1815)	694	402	63%	560	498	53%
*Canis lupus familiaris*	Dog (1897)	714	512	58%	627	581	52%
*Loxodonta africana*	Elephant (1485)	499	247	67%	428	312	58%
*Echinops telfairi*	Tenrec (1256)	368	179	67%	283	193	59%
*Takifugu Rubripes*	Fugu (203)	36	28	56%	24	28	46%
*Monodelphis domestica*	Opossum (1068)	249	169	60%	162	150	52%
*Dasypus novemcinctus*	Armadillo (1426)	469	260	64%	382	322	54%
*Gallus gallus*	Chicken (472)	113	36	76%	75	43	64%
*Danio rerio*	Zebrafish (207)	44	27	62%	26	32	45%
*Tetraodon nigroviridis*	Tetraodon (226)	46	24	66%	29	28	51%
*Xenopus tropicalis*	Frog (312)	74	37	67%	51	40	56%
**Human as a reference**							
*Pan troglodytes*	Chimp (575)	870	19	98%	867	15	98%
*Macaca mulatta*	Macaque (564)	800	53	94%	828	42	95%
*Mus musculus*	Mouse (488)	368	120	75%	364	105	78%
*Rattus norvegicus*	Rat (476)	369	112	77%	342	102	77%
*Oryctolagus cuniculus*	Rabbit (463)	445	86	84%	415	114	78%
*Bos taurus*	Cow (527)	531	122	81%	484	144	77%
*Canis lupus familiaris*	Dog (476)	546	121	82%	543	118	82%
*Loxodonta africana*	Elephant (458)	364	82	82%	341	83	80%
*Echinops telfairi*	Tenrec (419)	196	59	77%	175	68	72%
*Dasypus novemcinctus*	Armadillo (468)	362	95	79%	320	122	72%
*Monodelphis domestica*	Opossum (287)	213	35	86%	189	62	75%
*Gallus gallus*	Chicken (131)	33	10	77%	23	18	56%
*Takifugu Rubripes*	Fugu (80)	48	7	87%	51	11	82%
*Danio rerio*	Zebrafish (79)	43	7	86%	44	9	83%
*Tetraodon nigroviridis*	Tetraodon (87)	49	16	75%	52	18	74%
*Xenopus tropicalis*	Frog (89)	29	4	88%	29	10	74%

## Abbreviations

The following abbreviations are used in this manuscript:

lncRNA	long non-coding RNA
lincRNA	long intergenic non-coding RNA
nt	nucleotide
